# Patients' physical activity in stroke units in Latvia and Sweden

**DOI:** 10.1002/brb3.2110

**Published:** 2021-03-23

**Authors:** Agnese Kārkliņa, Erik Chen, Guna Bērziņa, Katharina Stibrant Sunnerhagen

**Affiliations:** ^1^ Department of Rehabilitation Riga East University Hospital Riga Latvia; ^2^ Department of Clinical Neuroscience Institute of Neuroscience and Physiology The University of Gothenburg Gothenburg Sweden; ^3^ Faculty of Rehabilitation Riga Stradiņš University Riga Latvia

**Keywords:** observation, physical activities, stroke rehabilitation, stroke unit

## Abstract

**Objective:**

A prospective, observational study to describe levels of physical activity in patients with stroke in a comprehensive stroke unit in Sweden and Latvia, comparing data between countries.

**Methods:**

The study was performed at stroke units in one hospital in Sweden (data were acquired over a 2‐month period in 2017) and two hospitals in Latvia (data were acquired over a 3‐month period between 2016 and 2017). Patients with stroke were observed for 1 min every 10 min. The level of physical activity, location, and the people present were noted at each time‐point.

**Results:**

A total of 27 patients were observed in Latvia and 25 patients in Sweden. Patients from both countries were in bed half of the time and spent the majority of the day in their bedroom and alone. Patients in Sweden had higher physical activity levels, spent more time outside their bedroom and spent more time with rehabilitation specialists and visitors.

**Conclusion:**

Patients are inactive and alone for a majority of the time during hospitalization at a comprehensive stroke unit in both countries. There are differences in environment in the stroke unit between countries.

## Graphical abstract

This behavioral mapping study describe levels of physical activity in patients with stroke in a comprehensive stroke unit in Sweden and Latvia. Patients are inactive and alone for a majority of the time during hospitalization in both countries. There are differences in environment in the stroke units between countries.

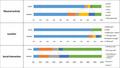

## 1 INTRODUCTION

Globally, stroke remains one of the most devastating of all neurological conditions (King's College London for the Stroke Alliance for Europe (SAFE), 2018). It is the second leading cause of death, affecting 15 million people annually, resulting in 5 million deaths and disability in 5 million people (Stroke Unit Trialists' Collaboration, 2013). While the majority of stroke patients survive, some patients are not able to fully recover their functional abilities (Cramer et al., 2017; Crichton, 2016) As a disease of aging, the prevalence of stroke is expected to increase significantly (King's College London for the Stroke Alliance for Europe (SAFE), 2018).

Organized inpatient care in stroke units, which is skilled nursing care and specialized rehabilitation, compared with general medical wards, increases the chance of survival and physical independence after stroke (Ayis et al., 2013; Stroke Unit Trialists' Collaboration, 2013). Early mobilization is one component of stroke unit care (Indredavik et al., 1999; Langhorne & Pollock, 2002). However, not all countries have stroke units and the content (organization, staffing etc.) differ between countries (Norrving et al., 2018).

During the acute phase of stroke, patients are inactive and alone (West & Bernhardt, 2012). Being inactive can lead to several medical complications such as pressure sores, bladder and bowel dysfunction, aspiration pneumonia, deep venous thrombosis, pulmonary embolism and falls (Bhalla & Birns, 2015; Harvey et al., 2015) and can also lead to deconditioning (Harvey et al., 2015). A lack of physical activity negatively affects the musculoskeletal, cardiovascular, respiratory, immune, metabolic and cognitive functions (Allen et al., 1999; Mutin‐Carnino et al., 2016), and can inhibit recovery after a stroke. Immobility related complications account for 51% of mortality within the first 30 days after the first ischemic stroke (Bamford et al., 1990). Preventing various complications caused by stroke is one of the major inpatient care and early rehabilitation interventions (Cardenas & Hooton, 2015; Jorgensen et al., 1999).

Stroke care is characterized by a continuity of process (Patrice et al., 2014) starting at the stroke unit where rehabilitation is initiated immediately after specialized treatment–reperfusion therapy thrombolysis or neurosurgery (European Stroke Organization, 2008) and continues until the patient returns to their usual environment (Ward et al., 2012). Any treatment that improves the functional outcome (functional independence ‐ improvement in mobility and activities of daily living) and can significantly reduce disability and costs, is important (Rosamond et al., 2008). Physical activities as out of bed activities or upright activities is an important factor stroke patient recovery (good outcome post‐stroke and better functional independence in the first months); higher levels of physical activity are associated with independence in daily activities (Billinger et al., 2014). Earlier studies show not only that the time since the stroke is important, but the dose and frequency of mobilization (physical activities) (Langhorne et al., 2017). Therefore, early rehabilitation should begin as soon as the patient's medical condition has stabilized (Stroke Unit Trialists' Collaboration, 1997).

The specific aims of this study were to compare levels of patients' physical activities as well as social interaction within stroke units in clinical university hospitals in Latvia and Sweden.

## 2 METHODS

### 2.1 Study design and duration

Prospective, observational, comparative (between countries) study using behavioral mapping. Data from Latvia were acquired over a 3‐month period between November 2016 and January 2017; data from Sweden were acquired over a 2‐month period between June 2017 and August 2017.

### 2.2 Pre‐specified data

#### 2.2.1 Baseline demographics

Patient demographics were extracted from medical records. Demographics that helped describe the patient and their stroke were recorded, this included age, gender, whether it is a first ever stroke, if the patient had aphasia after the stroke (patients with aphasia), mobility prior to stroke, specialized treatment of stroke, duration of stay in stroke unit. Neurological impairment was assessed with a National Institutes of Health Stroke Scale (NIHSS) score from the medical record (on the day of observation); patients were grouped into mild (NIHSS < 5), moderate (NIHSS 5–14), and severe (NIHSS ≤ 15) stroke categories (Brott & Adams, 1989). Disability prior to the current stroke event was estimated using the Modified Rankin Scale which was obtained from the medical records (on the day of observation) (mRS) (Van Swieten et al., 1988). NIHSS and mRS is done for reflecting patient's current status.

#### 2.2.2 Subjects and settings

The study was undertaken in two stroke units at two university hospitals in Latvia and in one stroke unit at a university hospital in Sweden. Patients (≥18 years) at a stroke unit with confirmed first or recurrent stroke (as defined by the World Health Organization; WHO) (Aho et al., 1980), at least 1 day (24 hr) post‐stroke were eligible for inclusion. Those patients who were receiving palliative care or were dying from their stroke were excluded. Also excluded were patients who had stroke due to subarachnoid hemorrhage. Approval for this study was obtained from Latvia (from both hospitals and from The Ethics Committee of Riga Stradiņš University; NR. 14/27.10.2016), from Sweden (Regional Ethics Board in Gothenburg, 384–17) and informed written consent was obtained prior to inclusion.

#### 2.2.3 Observational technique

Each patient was observed on a weekday from 9:00 a.m. to 3:00 p.m., i.e. the most active part of a patient's day. A standardized behavioral mapping form was employed, with observations every 10 min (from 9:00 a.m. till 2:50 p.m.), with the exception of a 20–30 min lunch break scheduled at different times in both countries. Each patient was observed for 1 min every 10 min. At the time of observation, the highest physical activity for a period (that is, in those 10 min) was recorded. On mapping forms at each time‐point an observer recorded patient activity, the person attending the patient, and their location (it counted as one observation). If feasible, patients were followed off‐ward, but not intruded on if in private situations (e.g., visiting the bathroom). However, once a patient was again visible, the patient or caregiver was questioned to determine the activity during the unobserved period. This observational method has been detailed previously (Bernhardt et al., 2004).

#### 2.2.4 Procedure

Patients and staff were informed that patients' activity throughout the day would be monitored, and it was emphasized that they should not do anything differently than what they would during a normal day. Eligible patients were informed and recruited to the study by a member of the local staff or by researcher. The unit was screened prior to the planned day of observation to determine whether observation was feasible. Mapping was considered feasible if a minimum of 1 and maximum of 8 in Latvia and minimum of 1 and maximum of 14 patients in Sweden were available on the day.

### 2.3 Professionals involved in the conduct of the study

In this study data collection was made by one person in Latvia and four people (medical students) in Sweden. The observation standards of the observers in Sweden were calibrated through common evaluation of several test subjects prior to the start of the study. Cohen's kappa coefficient was calculated between all four observers. The first five patients were observed in pairs and inter‐rater reliability of observed motor activity was calculated (O1/O2 *k* = 0.903; O2/O3 *k* = 0.802; O3/O4 *k* = 1.000; O4/O1 *k* = 1.000; Mean = 0.90, Range = 0.80–1.00, *SD* = 0.10). It is interpreted as almost perfect agreement (McHugh, 2012).

### 2.4 Categories of physical activity

One of fourteen whole‐body physical activities was recorded at each observation. Those 14 activities were grouped into five pre‐specified activity categories (ACs), to reflect the level of physical work during these activities. The categories have been classified by experienced clinicians and used in previous behavioral mapping studies (Bernhardt et al., 2004, 2008). Observed motor activity and activity categories are represented in Table 1.

**TABLE 1:** Categories of physical activities

Activity category	Activity class	Observed activity
AC0	No activity	No motor activity
		Transfer with hoist
AC1	Non‐therapeutic activity	Eat in bed supine
		Talk, watch TV, read in bed supine
AC2	Minimal therapeutic activity	Sit supported in bed
		Sit supported out of bed
AC3	Moderate therapeutic activity	Roll and sit up
		Sit unsupported
		Transfer feet on floor
AC4	High therapeutic activity	Standing
		Walking
		Walking a stairs

### 2.5 Social interaction and location of activity

People present were recorded if they were interacting with the patient. Eating at different tables or sharing a room did not automatically count as interaction. The nine categories for people present were recorded as: medical staff as doctors (and other medical team members), nurses, nurse assistants, occupational therapists (OT), physiotherapists (PT), speech therapists (ST), family (visitors), others (as other patients, patient transporter, and social worker), alone. The six categories for location were bedroom, hall, bathroom/toilet, therapy area, patients' lounge and off‐ward.

### 2.6 Data processing and analysis

The highest level of activity (5 ACs) in every 10‐min interval was recorded in the database (Microsoft Excel). Results are expressed in relative numbers as a function of time, 100% being equal to a behavior lasting a 6‐hr day (9:00 a.m.–3:00 p.m.).

Statistics were used to describe:

1. Data on patients: age (years) and duration of stay in the stroke unit (days), mean (M), standard deviation (*SD*), median (Me), minimum (Min), maximum (Max) was reported. First stroke, NIHSS, mRS, aphasia, mobility prior to stroke, specialized stroke treatment, as well as data on physical activity and social interaction were reported in proportions for Latvia and Sweden. Comparison between countries were made by Mann–Whitney *U* test and Chi‐square test for continuous and categorical data respectively. To compare differences between patients in Latvia and Sweden independent *t*‐tests were used.
2. Data on stroke units: qualitative comparison of stroke units.

There result was considered as statistically significant if the *p* value was less .05. All statistical analyses were performed with IBM SPSS statistics version 22.0.

## 3 RESULTS

In the Latvian population 1815 observations were made with 27 patients whereas in Sweden 1,202 observations were made with 25 patients. Comparison of patient characteristics and stroke units between Latvia and Sweden are presented in Tables 2 and 3 respectively.

**TABLE 2:** Patient's characteristics: Comparison between Latvia and Sweden

Patient's characteristics	Latvia *n* = 27	Sweden *n* = 25	*p* *
Age, years, mean (*SD*), median [range]	71.3 (15), 76 [18–90]	72.4 (16.4), 76 [27–90]	.49
First‐ever stroke, *n*	23	23	.44
National Institutes of Health Stroke Scale (NIHSS), *n*			
Minor stroke (1–4)	6	17	**.01**
Moderate stroke (5–14)	14	7	
Moderate to severe stroke (15–24)	7	1	
Modified Rankin Scale (MRS) prior stroke, *n*			
No to slight disability (0–2)	3	19	**.00**
Moderate to severe disability (3–5)	24	6	
Patients with aphasia, *n*	15	7	.16
Pre‐stroke mobility, *n*			
Independent no aids	19	19	.40
Independent with aid	5	0	
Dependent	3	6	
Reperfusion treatment, *n*			
Thrombolysis	11	5	.26
Thrombectomy	3	1	
Duration of stay in Stroke unit (in days), mean (*SD*), median, [range]	4.6 (1.1), 4.0, [3–7]	8.2 (6.1), 6.0, [2–23]	.29

* Bold value indicates statistically significant differences between countries if *p* ≤ .05.

**TABLE 3:** Characteristics of stroke units: comparison between Latvia and Sweden

Characteristics of stroke units	Latvia *n* = 2	Sweden *n* = 1
Type of stroke unit	Multidisciplinary	Multidisciplinary
Number of beds	16 (8 in each)	14
The average length of stay (in days)	Maximum 5 days (for these patients average time was 4.56 days)	No limit (for these patients the average time was 8.16 days)
Doctor: patient ratio	2 doctors and 2 residents: 8 in each^a^	2 doctors and 2 residents: 14
Nurse: patient ratio	1:8/2:8	3:14
Nurse assistant: patient ratio	1:8/2:8	2:14
Patient transport: patient ratio	1:8^a^	No data available
PT: patient ratio	2:8^a^	6:14
OT: patient ratio	2:8/1:8^a^	4:14
ST: patient ratio	2:8/1:8^a^	0.5:14
Social worker	On consultant basis^a^	Part time^a^
Psychologist	0	On consultant basis^a^
Therapy room on‐site (speech therapy, physiotherapy and occupational therapy)	Differs between hospitals: training room for PT, PT and ST on ward or in another floor. No specific training rooms available.	Training room for PT on the ward, training room for OT on the ward. More specific training rooms such as kitchen training require that the patient and OT go to another building.
Dining area	In rooms (in beds) special table for each patient	Dining area and possibility to eat in the rooms as well
Patient's lounge	Lounge in hall, no TV available	TV in the rooms and also in the dining area
Rehabilitation staff working time	Therapists work a 5‐day week (8−16/17), no work on holidays	Therapists work 5‐day week (7.30–16.15) 1 PT and 1 OT are available on Saturday (8–13)

Abbreviations: OT, occupational therapist; PT, physiotherapist; ST, speech therapist.

^a^ Those specialists who were working elsewhere in hospital (not only in Stroke unit).

**FIGURE 1:**
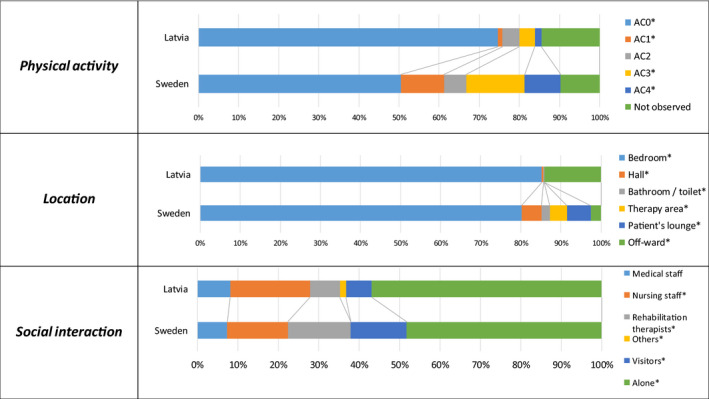
Comparison of patient's physical activity levels, location and social interaction between Latvia and Sweden observed between 09.00 hand 15.00 hr. *p ≤ .05 comparing between two countries

Comparison of physical activity levels, patient's location and social interaction between Latvia and Sweden see in Figure 1.

Looking at physical activity levels, AC0 (had no activity) level time was 75% (4.5 hr) for patients in Latvia whereas in Sweden it was 50% (3 hr). In Sweden patients had AC1, AC2, AC3, AC4 activities 40% of time, but only 11% in Latvia. For example, AC4 (high therapeutic activity) in Sweden was 9%, but 1.6% in Latvia. Most of the levels differed statistically significantly between countries (*p* = .05) except AC2 (*p* = .49), which is minimal therapeutic activity (sit supported in bed; sit supported out of bed), and time, which was not observed (*p* = .19).

Most of the time during the day, from 9:00 a.m.−3:00 p.m., patients were in their bedroom ‐ in Latvia it was 85% of time (5.1 hr), but 72% (4.3 hr) in Sweden. In Latvia time spent in the hallway, bathroom, therapy area and patients' lounge was about 0.6%, whereas in Sweden it was 16%. In Latvia patients were off‐ward for about 14% of the time, compared to 2% in Sweden. Data differed statistically significantly for all locations (bedroom *p* = .01; hall *p* = .00; bathroom *p* = .00; therapy are *p* = .00; patients' lounge *p* = .00; off‐ward *p* = .00).

Patients spent most of the time from 9:00 a.m.−3:00 p.m. alone–in Latvia it was 57% of the time (3.4 hr), but in Sweden it was 48% (2.9 hr) (*p* = .025). In Sweden patients spent more time with rehabilitation therapists (16% versus 7% in Latvia) (*p* = .00) and visitors (14%, 6% in Latvia) (*p* = .01). Nursing staff spent 20% of time with patients in Latvia, but 15% in Sweden (*p* = .03). No statistical differences were found between the countries regarding the time medical staff interacted with patients (*p* = .60).

## 4 DISCUSSION

The results of this study confirm that patients spent the most active part of the day inactive and alone in clinical university hospitals both in Latvia and in Sweden. However, the physical activity level, social interaction, and locations differ significantly between those two countries. This is the first study mapping physical activities in the acute phase of stroke in a stroke unit that compares data of two countries.

The study included three stroke units that provide multidisciplinary stroke treatment, including rehabilitation professionals, which has been shown to be the best form of stroke treatment and care (Stroke Unit Trialists Collaboration, 2013; Sun et al., 2013). As mentioned previously, physical activity levels are probably dependent on the environment at the stroke unit. Describing the physical environment in both Stroke units in Latvia, it should be noted that in both hospitals there are factors that limit physical activity. Most basic activities for patients are provided within their bedroom. For example, food is brought to the patients in bed, patients eat on the ward (half‐sitting with support in bed or sitting in bed with or without support). This is a classic approach in hospitals and does not encourage physical activity. One study found that stroke units patients were more physically active if food was served in communal areas compared with units in which food was served in patients' rooms (Hokstad et al., 2015). There are several theories that suggest that the environment promotes or hinders the pursuit of an activity: it drives an individual's actions with certain requirements and limitations, providing opportunities and resources for the individual's actions (Law et al., 1996; Lawton & Nahemow, 1973). In one study, patients in a stroke unit were observed through reconstruction of the physical environment (Anåker et al., 2017). The findings indicated that in the new unit, the patients spent more time in their rooms, were less active, and had fewer interactions with staff and family than the patients in the original unit (Anåker et al., 2017). The reconstruction involved a change from a primarily multi‐bed room design to single‐room accommodations (Anåker et al., 2017). In the new unit, the patients' lounge was located in a far corner of the unit with a smaller entrance than the patients' lounge in the old unit, which was located at the end of a corridor with a noticeable entrance. Changes in the design of the stroke unit may have influenced the patients' activities and interactions (Anåker et al., 2017). It is possible to talk about finding environmental solutions that do not contradict the existing norms in the hospital, but would also promote the patient's activity (for example, a lounge and a separate room for eating in Latvia as already exists in the Swedish stroke unit).

One of the additional aspects that explain low patient activity is the lack of private space. When a patient is in hospital, the only private place available to him/her is a bed in which he/she may feel more psychologically secure. In the stroke units in Latvia, all the beds are in one room with no single rooms available. However, in this particular Swedish context, there are single rooms, double room, and triple rooms depending on the medical and psychological status of the patients. Patients in Latvia are not allowed to go to the patients' lounge alone (due to of risk of falls etc.), patients need to sleep or sit in bed so that nursing staff can observe them.

The data from this study are similar with data from other studies that provide information on patients' activity in the acute phase after a stroke: patients spend most of the day inactive and alone in a stroke unit (Harvey et al., 2015; West & Bernhardt, 2012). A higher NIHSS score is associated with lower levels of physical activity (Bernhardt et al., 2004). Although NIHSS differed statistically in this study, patients in both Latvia and Sweden were inactive for approximately half time from 9:00 a.m.−3:00 p.m. Patients in Sweden were more active.

In the literature, social interaction has been described as important for promoting activity in stroke patients (Janssen et al., 2014). Time spent with nursing staff, rehabilitations specialists, visitors, and alone differed statistically between countries. In Sweden, time spent with rehabilitation specialists was 50% more than in Latvia. It may be related to different staffing ratios.

Family should also be regarded as a resource for activating patients (Skarin et al., 2013). It should be mentioned that one hospital in Latvia has a limited visit time (from 1:00 p.m. to 3:00 p.m.), while in the other hospital in Latvia ‐ patient visit time is not limited, but there was a flu epidemic in the middle of data collection, so restrictions for visitors were made. In Sweden visitor time was not limited. Because living with a family member is associated with returning home, involving family in early rehabilitation might be important (Van de Port et al., 2012).

### 4.1 Methodical considerations and limitations

Limitations of this study include having a small number of patients; it is possible that with a larger number of patients and more observations, the results of the study would be different. The research was carried out by including stroke units in Riga, excluding units in the periphery, which thus limits a more complete study of the situation in stroke units in Latvia, and included only one stroke unit in Sweden. On the other hand, they all are stroke units in clinical university hospitals which reflects the standard for care in each country. Sample size was not calculated for this study, only the time frame was set. There is a probable selection bias, because the most part of the patients in this study had a minor stroke in Sweden, but a moderate stroke in Latvia. In this study, patients were observed on weekdays from 9:00 a.m. to 3:00 p.m., which is the most active time of the day in the hospital (West & Bernhardt, 2012). There is no information on patients' physical activity and social interaction on weekday evenings and weekends. Physical activity mapping studies, in which patients were observed throughout the working day (including evenings) and on weekends, concluded that compared to the active time of the working day (8:00 a.m. to 5:00 p.m.), patients were still less active; similarly, in many stroke units, rehabilitation staff only work on weekdays, which means that rehabilitation is not provided to patients on weekends or holidays (De Wit et al., 2005; Mackey et al., 1996; Tinson, 1989). Another limitation of the study is the possible subjectivity of the observed data collection in Latvia, as only one researcher observed the patients and collected the data. It is possible that staff or patients changed their behavior due to the presence of an observer. The researcher avoided encouraging or restricting the activity of patients and staff in order to observe real practice as much as possible. Also, there are other methods and devices to monitor patients' physical activities, for example, accelerometers (Fini et al., 2015), however, these have technical problems and do not give the information on content or social components.

The adjustment for factors were considered since severity of stroke and levels of independence were significantly different between the Latvian and Swedish cohorts. However, for reporting proportions of physical activity levels, summary population scores were used and no statistical adjustment was possible. Yet, these differences does not explain, why time spent with rehabilitation specialists is Sweden was 50% more than in Latvia.

No information was collected about other comorbidities which can affect patient's functional ability and cooperation. In this study, the organizational aspects rather than functioning characteristics were analyzed as factors influencing the activities. However, in a future study, comorbidities also should be included.

Levels of physical activity in stroke patients do not meet the guidelines for normal physical activity ‐ time spent inactive and sedentary is high. There is a wide range of opportunities for patients in the stroke unit to be more physically active with the support and assistance of nurses and nursing assistants, the involvement of rehabilitation professionals and visitors in promoting activities. Increasing physical activities and developing standardized activity targets may be important across the acute phase of stroke (24 hr post‐stroke).

In a study performed by Anåker et al., (2017) the question of how the physical environment should be designed in the future to facilitate the delivery of health care and improve outcomes for stroke patients was raised and they strongly recommended that environmental considerations be included in future stroke guidelines. The created physical environment should stimulate patients' activities (enriched environment) (Anåker et al., 2017).

## 5 CONCLUSION

Patients are inactive and alone for a majority of the time during hospitalization at a comprehensive stroke unit in both countries. There are differences in environment in the stroke unit between countries. There is a potential to promote physical activity levels above current levels and create enriched environment.

## CONFLICT OF INTEREST

The authors have no conflicts of interest to declare.

## AUTHOR CONTRIBUTIONS

AK performed data collection in Latvia, was responsible for data analysis, interpretation and writing. EC performed data collection in Sweden and was involved in the interpretation of the results. GB and KSS were involved in study design and interpretation of the results. All authors have read and approved final manuscript.

### PEER REVIEW

The peer review history for this article is available at https://publons.com/publon/10.1002/brb3.2110.

## Data Availability

The data from this study are available on reasonable request from the corresponding author.
